# Web-based infectious disease surveillance systems and public health perspectives: a systematic review

**DOI:** 10.1186/s12889-016-3893-0

**Published:** 2016-12-08

**Authors:** Jihye Choi, Youngtae Cho, Eunyoung Shim, Hyekyung Woo

**Affiliations:** 1 Department of Public Health Science, School of Public Health, Seoul National University, 1 Kwanak-ro, Kwanak-gu, Seoul, South Korea; 2 Department of New Business, Samsung Fire and Marine Insurance, 14 Seocho-daero 74-gil, Seocho-gu, Seoul, South Korea

**Keywords:** Surveillance systems, Epidemics, Outbreak, Real-time, Web-based, Early detection

## Abstract

**Background:**

Emerging and re-emerging infectious diseases are a significant public health concern, and early detection and immediate response is crucial for disease control. These challenges have led to the need for new approaches and technologies to reinforce the capacity of traditional surveillance systems for detecting emerging infectious diseases. In the last few years, the availability of novel web-based data sources has contributed substantially to infectious disease surveillance. This study explores the burgeoning field of web-based infectious disease surveillance systems by examining their current status, importance, and potential challenges.

**Methods:**

A systematic review framework was applied to the search, screening, and analysis of web-based infectious disease surveillance systems. We searched PubMed, Web of Science, and Embase databases to extensively review the English literature published between 2000 and 2015. Eleven surveillance systems were chosen for evaluation according to their high frequency of application. Relevant terms, including newly coined terms, development and classification of the surveillance systems, and various characteristics associated with the systems were studied.

**Results:**

Based on a detailed and informative review of the 11 web-based infectious disease surveillance systems, it was evident that these systems exhibited clear strengths, as compared to traditional surveillance systems, but with some limitations yet to be overcome. The major strengths of the newly emerging surveillance systems are that they are intuitive, adaptable, low-cost, and operated in real-time, all of which are necessary features of an effective public health tool. The most apparent potential challenges of the web-based systems are those of inaccurate interpretation and prediction of health status, and privacy issues, based on an individual’s internet activity.

**Conclusion:**

Despite being in a nascent stage with further modification needed, web-based surveillance systems have evolved to complement traditional national surveillance systems. This review highlights ways in which the strengths of existing systems can be maintained and weaknesses alleviated to implement optimal web surveillance systems.

**Electronic supplementary material:**

The online version of this article (doi:10.1186/s12889-016-3893-0) contains supplementary material, which is available to authorized users.

## Background

Despite medical advances and increased vaccine availability, emerging and re-emerging epidemics continue to pose tremendous threats, based on reported cases of severe acute respiratory syndrome, influenza A (H1N1), avian flu, Ebola virus, and the recent Middle East respiratory syndrome [1]. To avoid the repercussions of an epidemic, early detection and immediate response are emphasized to manage infectious diseases. Many online surveillance systems that function based on real-time data have been developed involving a wide range of technologies and data sources to prevent the occurrence of infectious diseases; these systems are continually being added to and evaluated [2]. Traditional passive surveillance systems typically rely on data submitted to the relevant public health authority by various healthcare providers [3]. This process is often expensive and inefficient, as substantial delays between an event and notifications are common, resulting in an incomplete account of disease emergence. Such limitations of traditional surveillance systems are a shared concern worldwide.

The Internet has revolutionized efficient health-related communication and epidemic intelligence [4]. The increased frequency of Internet use for acquiring health information has contributed to the rise of web-based early detection systems for infectious diseases through various methodologies [5]. The principal concept is that disease-related information is retrieved from a wide range of available real-time electronic data sources, which play critical roles in the identification of early events and situational preparedness by offering current, highly local information about outbreaks, even from remote areas that have been unapproachable by traditional global public health efforts [6]. These systems not only monitor and predict disease outbreaks but also provide a user interface, and aid in visualization for an easier understanding and maneuvering of the operation. These new systems for early detection of epidemics are still in the nascent stage, but the concept and relevant promising mechanisms have been adopted and tested by the Centers for Disease Control and Prevention (CDC) with positive indications for efficiency and feasibility [7]. In fact, several web-based surveillance systems are affiliated with the CDC from which they are granted funding and technical assistance [8].

Previous studies have suggested that these new systems exhibit remarkable potential for expansion and for enhancing the capacity of traditional surveillance systems for emerging infectious diseases [9]. It is of great importance to discuss the possible directions in which these new surveillance systems are headed in the context of public health by thoroughly examining areas of improvement for such systems. In addition, the absence of a system for predicting and monitoring epidemics in some countries with strong information communications technology (ICT) capability should command the attention of their national public health sectors, as there is an imminent need to implement such a mechanism. The objective of this systematic review was to investigate well-established web-based infectious disease surveillance systems that focus on infectious disease occurrence and the early detection of outbreaks. Our investigation can serve as an overview and starting point for readers interested in the topic and as a useful reference for the design of prospective infectious disease surveillance systems in countries that lack such tools.

## Methods

A systematic review was performed and reported in accordance with the Preferred Reporting Items for Systematic Reviews and Meta-Analyses checklist (Additional file 1).

### Eligibility criteria and information sources

Literature from multiple journal sources was obtained by searching with relevant search terms, and appropriate articles on web-based disease surveillance systems were reviewed extensively. The literature search was conducted using the PubMed, Web of Science, and Embase databases. Articles written in English published between 2000 and 2015 were searched for a more refined outcome. The following key words were used in the search process: syndromic surveillance (“syndromic” [All Fields] AND “surveillance” [All Fields]), digital disease detection (“digital” [All Fields] AND “disease” [MeSH Terms] AND “detection [All Fields]), biosurveillance (“biosurveillance” [MeSH Terms]), infoveillance (“infoveillance” [All Fields]), infodemiology (“infodemiology” [All Fields]), online surveillance (“online” [All Fields] AND “surveillance” [All Fields]), outbreak forecast (“outbreaks” [All Fields] AND “forecasting” [MeSH Terms], and web surveillance systems (“web” [All Fields] AND “surveillance” [All Fields] AND “systems” [All Fields]). The initial search strategy developed for PubMed was that some of the vague terms were re-sorted into “medical subject headings”, which brought forth more specific and relevant results.

### Study selection process

**Fig. 1:**
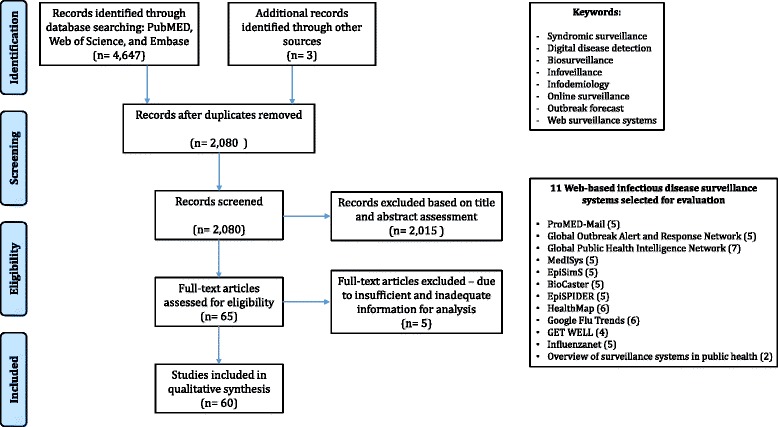
Flowchart of the systematic review process

The first task was to systematically search the three databases PubMed, Web of Science, and Embase. Second, the 4,650 articles identified after the removal of duplicates were meticulously checked for relevant information on web-based infectious disease surveillance systems. Third, those web-based infectious disease surveillance systems which were mentioned in at least five studies were further considered. Lastly, all identified evidence was further complemented with the authors’ expert knowledge and personal archives. The last step also included the consultation of the CDC website and the inclusion of the “GET WELL” system, which was only mentioned in four studies (see Fig. 1) and would have been omitted without this last step. Other web-based infectious disease surveillance systems that were mentioned in only a few studies and thus were not considered in this systematic review are as follows: Argus, Electronic Surveillance System for the Early Notification of Community-based Epidemics (ESSENCE II), International system for Total Early Disease Detection (InsTEDD). The studies included provided a comprehensive review for understanding existing web-based surveillance systems aimed at detecting infectious diseases early.

Typical terms associated with conventional systems have evolved following the emergence of new online-based infectious disease surveillance systems. The merging of public health and ICT has brought forth several recently coined terms and unprecedented word combinations, both of which are essential for understanding the fundamentals of the new disease detection systems. These new terms reflect the complexity of the convergence. The most commonly appearing terms and their descriptions are shown in Table 1.

**Table 1:** Newly coined terms in web-based surveillance systems and their descriptions

Term	Description
Syndromic surveillance	The systematic process of data collection and analysis to detect and characterize disease outbreaks in humans and animals in a timely manner [10, 11].
Biosurveillance	The process of gathering, integrating, interpreting, and communicating essential information that might be related to activity and threats to human, animal, or plant health [12]. Biosurveillance activities range from standard epidemiological practices to advanced technological systems, utilizing complex algorithms [13]. The focus is on the use of early disease indicators to identify outbreaks before definitive diagnoses are made [14].
Infodemiology	Information epidemiology; the science of the distribution and determinants of information in an electronic medium, specifically the Internet, or in a population, with the ultimate aim to inform public health and public policy [15, 16].
Infoveillance	Information surveillance; longitudinal tracking of infodemiology metrics for surveillance and trend analyses [16–18].
Digital surveillance	Attempts to provide knowledge of public health issues by analyzing health information stored digitally, as well as the distribution and patterns governing access to these data [18].
Real-time surveillance	Encompasses alerting public healthcare practitioners during the early phases of an outbreak, enabling them to promptly institute control measures and case finding and to ensure adequate access to treatment, thereby reducing morbidity and mortality [19].

## Results

### Numbers of articles identified and web-based surveillance systems further considered

Across the three databases and the CDC website, 4,650 articles were collected, and duplicates were removed within the same database and across the different databases, resulting in 2,080 articles. Subsequently, these articles were further screened by assessing whether the title or abstract contained the exact search terms or if the content itself was relevant to the subject matter. After a meticulous assessment of full-text articles for eligibility, and exclusion of those with insufficient and inadequate information for analysis, 60 studies were filtered for the final qualitative analysis. Eleven web-based surveillance systems were analyzed, based on the selected literature, with regard to their development, various characteristics, and mechanisms, including their methods of data collection and delivery of service. The flow chart (see Fig. 1) illustrates the literature selection process for this systematic review.

### Development of web-based surveillance systems

As newly emergent and resurgent infections have progressively become a significant threat to the global community, a more systematic approach is needed to respond to these challenges [12]. Web-based reporting and surveillance systems first originated to strengthen global capacity for disease surveillance [20]. The forerunner was the Program for Monitoring Emerging Diseases (ProMED-Mail), which was established in 1994 under the auspices of the Federation of American Scientists, with the aim of rapidly disseminating disease-related information to a wide audience and allowing for informed discussion in real-time. However, it has been operated by the International Society for Infectious Diseases since 1999 [21]. Subsequently, the World Health Organization (WHO) established an effectively organized infrastructure called the Global Outbreak Alert Response Network (GOARN) for the very first time, which served as a “network of technical partners and other networks with the capacity and expertise to contribute to an international coordinated response to outbreaks of epidemic-prone and novel infectious diseases” [22].

**Fig. 2:**
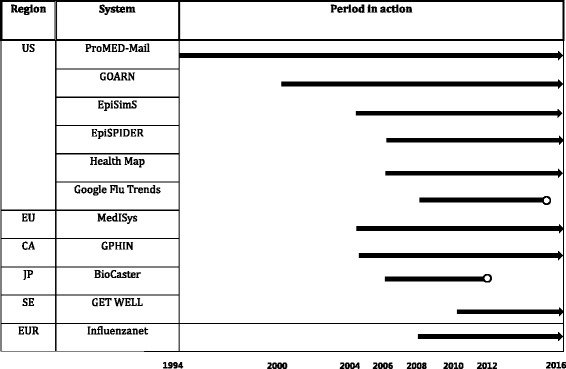
Development of surveillance systems in chronological order

Following the information revolution and the rise of web 2.0, active and frequent use of the Internet triggered the creation of more surveillance systems [5]. While earlier network-based infrastructure focused on news reports as the primary data source, recently created surveillance systems use various sources for early warning systems, developed in several countries, which include query data from online search engines and social media such as Twitter [23]. Moreover, some Internet-based surveillance systems have been selected to be part of a national security system and are managed at the national level. Such a phenomenon is most often apparent in developed countries, as in the United States and Sweden. CDC funds feasible and effective surveillance systems to enhance the technical aspect, and the Generating Epidemiological Trends from Web Logs, Like (GET WELL) system has been officially accepted by the Swedish government and is in regular use at the Swedish Institute for Infectious Disease Control, providing a complementary aid to the daily surveillance performed by epidemiologists [24]. Over the last decade, these systems have progressed dramatically, as evidenced by the transformation in data collection and dissemination (Fig. 2).

### Data source and logic of web-based surveillance systems

**Fig. 3:**
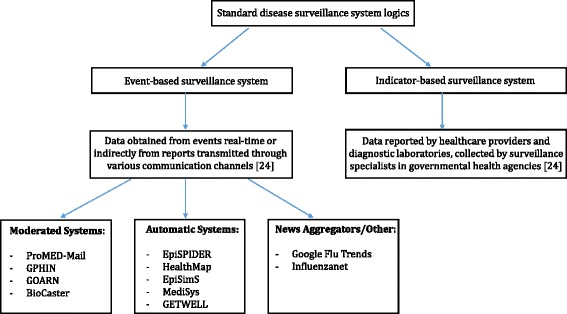
Classification of standard disease surveillance systems

Web-based surveillance systems have been developed to monitor news reports and to rapidly spread information on disease outbreaks with the aim of detecting an infectious disease at the onset of the outbreak. Figure 3 shows the classification of standard disease surveillance systems. Event-based surveillance systems are based on the organized, rapid capture, and reporting of information about outbreaks or events that can be a risk to public health [25–27]. However, rather than relying on official reports, this information is retrieved directly from witnesses of real-time events or indirectly from reports transmitted through various communication channels, such as social media, and information channels including news media and public health networks [28]. A great deal of attention from the public, and media interest, are associated with an epidemic [29, 30]. Health information monitored via the Internet and social media is a pivotal part of event-based surveillance and is most often the source emphasized by many existing surveillance systems [18]. Event-based disease surveillance systems can be classified into three main categories of news aggregators, automatic systems, and moderated systems. Moderated systems function so that information is processed by human analysts or is processed automatically before being analyzed by human analysts [31, 32]. These systems screen for epidemiological relevance of the data extracted within the information prior to being presented to the user [26]. Examples of this system include ProMED-Mail, GPHIN, GOARN, and BioCaster. The process by which automatic systems collect data is complex; it adds a series of steps for analysis, but differs in the levels of analysis performed as well as in the scope of information sources, language coverage, speed of delivery, and visualization methods. EpiSPIDER, HealthMap, EpiSimS, MedISys, and GETWELL are examples of automatic systems [33–35]. Finally, news aggregators include Google Flu Trends, which collect reports and articles from sources screened by language or country; by such means users can easily access many sources via a common portal but they are required to view each article individually [26].

### Delivery of service

Most new surveillance systems have been applied worldwide, as seen through the structured table of the systems categorized according to the origin, area of service, language coverage, data source, data access, user interface and format and arte offered in different languages, except EpiSimS and GET WELL [36]. BioCaster, EpiSPIDER, and HealthMap are disseminated on a geographical map. MedISys and ProMED-mail are disseminated through websites or news aggregators in the public, whereas GOARN and GPHIN are disseminated through a secured or restricted portal accessed by entities with monitoring responsibility, who respond to and mitigate emerging public disease threats [37, 38]. Influenzanet is a unique system, as it obtains data directly from the population; this participatory system monitors the activity of influenza-like illnesses in real-time with the aid of volunteers with certain symptoms and via internet questionnaires comprised of various medical, geographic, and behavioral questions [39, 40]. Table 2 below summarizes the various characteristics of 11 of the most often used and/or recognized web-based surveillance systems.

**Table 2:** Organization of the surveillance systems by various characteristics

System	Country (Year started)	Area of Service	Language	Data Source	Data access	User Interface	Format
ProMED-Mail	USA (1994)	Worldwide [21]	7 languages [26]	News/Media Report	Public	None	E-mail alert [37]
GPHIN	Canada (1997, 2004^a^)	Worldwide	8 languages [26]	News/Media Report (Factiva and Al Bawaba) [41]	Restricted/Subscription [22]	Boolean and metadata query system [35]	Website/e-mail alert [22]
GOARN	Multiple^b^ (2000)	Worldwide	English, multilingual [26]	News/Media Report	Restricted [26]	None	Network-based
MedISys	EU (2004)	European Union member states [42]	43 languages [26]	News/Media Report [42]	Restricted/Limited to EU member states [42]	Text extraction [43]	RSS feed/E-mail and SMS alerts [44]
				Europe Media Monitor			
EpiSimS	USA (2005)	United States	English	US Census data [45]	Restricted	Graphical user interface; simulation pre-processing [45–48]	XML-based format
				Transformation infrastructure data			
BioCaster	Japan (2006)	Priority to Asia-Pacific region [33]	8 languages [26]	Query	Public [49]	Mapping interface [13]	RSS feed [13]
				Text mining [13]			
EpiSPIDER	USA (2006)	North America, Europe, Australia, Asia	English [26]	News/Media Report	Public	Timeline visualization mapping and word cloud [33]	RSS, JSON, KM feeds [6, 50]
				Social Media			
				CIA [51]			
Health Map	USA (2006)	Worldwide	5 languages [26]	Query	Public	Mapping, faceted browsing (native) [52]	RSS feed [18]
				News/Media Report [52]			
				ProMED, WHO, Euro Surveillance [53]			
Google Flu Trends	United States (2008)	28 countries [54]	39 languages [54]	Query [55]	Public [56]	Mapping features [18]	RSS feed [18]
				CDC [57]			
GET WELL	Sweden (2010)	Sweden	Swedish	Query [24, 58]	Restricted [24]	Time-series graphs [24]	HTML page [36]
Influenzanet	Europe (2008)	The Netherlands, Belgium, Portugal, Italy, the UK, France, Sweden, Spain, Ireland, Denmark, Switzerland [59]	10 languages	Self-report from volunteer (online questionnaire response) [39]	Public [60]	None	RSS feed [61]

^a^GPHIN was first established in 1997 but a new, robust multilingual GPHIN system was developed and launched on November 17, 2004 at the United Nations [22]

^b^GOARN is a network collaboration between the World Health Organization and the United Nations

## Discussion

### Evolution of research on web-based infectious disease surveillance systems

The development of and access to telecommunications, media, and the Internet marked the starting point for implementing web-based surveillance systems. The vast majority of surveillance systems were developed simultaneously from 2004 to 2006. An unprecedented increase in the number of Internet users was observed during this period, followed by growth of social network services and the introduction of big data. These changes were sufficient to spark integration between the ICT and public health issues, leading to the rise of web-based disease surveillance systems. The first systems were regarded as pilot trials at the exploratory level, and were often based at, or in cooperation with, universities or institutions (BioCaster, HealthMap, and GETWELL), non-governmental organizations (GOARN, MedISys, and ProMED-Mail) and a few governmental agencies (EpiSPIDER and GPHIN). Since the initiation of these web-based surveillance systems as trial programs, many have evolved and become renowned over the past few years.

Several general trends are observed among the characteristics of the 11 web-based surveillance systems. Most of the web-based surveillance systems were first developed in North America, particularly the United States, with abundant infrastructure and technological resources, when integration of ICT and syndromic surveillance for early detection and response to diseases was at a preliminary phase. As time progressed, other regions, such as Asia and Europe, have caught up by launching similar but distinct web-based surveillance systems, spreading the notion of early detection of disease outbreaks by real-time scanning and collecting, and analyzing unstructured information from diverse internet sources [62]. English was the only language in service in the earlier systems but, subsequently, the collection and analysis of data began to be published in different languages based on the service area. The scope of data sources has also expanded as newer surveillance systems extract information not just from secondary news reports but also from social media, web search queries, and various organizations such as the CDC, Central Intelligence Agency, and the WHO.

The terminology has changed among the many elements of the web-based surveillance systems that have evolved and become sophisticated. The fusion of epidemiologic intelligence and ICT has produced newly coined terms that describe the core functions and characteristics of web-based surveillance systems. This new terminology is essential for depicting the underlying importance of digital technology as a public health tool. Future web-based surveillance systems will produce additional new terms to highlight the collaborative characteristics of these systems.

The best recognized use of novel technologies and health surveillance data together is that of estimating the range and magnitude of health problems in a community to rapidly detect the outbreak of an epidemic at its onset [63]. It is evident that web-based surveillance systems have huge potential to enhance traditional systems, as opposed to merely being an alternative, as they have added benefits and capacities, such as a large quantity of relevant data, increased accessibility, and timeliness [63, 64].

### Strengths and future challenges of newly emerging surveillance systems

Internet-based systems are intuitive, adaptable, inexpensive to maintain, and operate in real time [3]. Advanced computational capabilities involving Internet searches enable automated and rapid collection of large volumes of data, referred to as “big data”, and provide the public with “real-time” detection and improved early notification of localized outbreaks [65]. In addition, a system based on web queries can easily be applied to various infectious diseases, as the underlying mechanisms are very similar [66].

Some groups, such as the WHO, CDC, and other governmental and multi-lateral bodies, have begun to recognize the added value of these tools through the use of technologies, such as HealthMap and other new initiatives [52, 67]; such acceptance serves as a valuable lesson for developing countries shaping the future of their public health systems. Developing countries that are particularly prone to the spread of infectious disease should seek ways to emulate the strengths of existing web-based surveillance systems and broaden the group of users directly accessing and utilizing such systems [68].

However, the new Internet-based surveillance systems are not without limitations, thereby provoking skepticism. First, due to the unstructured nature of the data sources, interpreting the information may require highly complex techniques to effectively implement the system initially [69]. The recent closure of Google Flu Trend was partially due to its failure to provide a swift and accurate account of flu outbreaks [70]. Although the quantity of information was thought to be reliable for monitoring and predicting the occurrence of a flu outbreak, the lack of methodological transparency for data extraction, processing, and analysis led to inaccurate prediction in detecting an influenza outbreak [71]. Second, Internet use and health-seeking behavior vary among individuals, and between different sectors of the community and environment. Thus, the limited environments in which these tools are useful must be considered along with the demographics of the population [72]. Large discrepancies occur between availability of the Internet and active seeking of healthcare information that account for unequal use and access [73, 74]. Third, data sharing permits more and better quality data to be used to monitor public health and potential outbreaks [75]. However, use of data with precise information connected to individuals could be a privacy concern. Careful and appropriate decisions need to be made to avoid any further privacy intrusion on personal information. Last, forecasting health and disease-related phenomena is very likely to provoke accuracy issues because health fluctuates in every individual, and how people perceive their health status is very subjective. Although monitoring trends in disease outbreaks and health outcomes is possible, forecasting them is subject to false predictions. Thus, data sources must be evaluated extensively, particularly to identify gaps in coverage and false decisions [76]. The expectation now is that the accuracy of these systems will be enhanced through iterative procedures and that the scope of search-term surveillance will be more inclusive to other diseases [69]. The precedent of the Google Flu Trend failure illustrates the importance of a balance between traditional data and big data to maintain these systems. It is probable that future challenges will remain with regard to data integration, compatibility issues, and evaluating surveillance systems, all of which are underdeveloped and lacking in the current research. More research addressing these issues will be necessary.

### Considerations and recommendations for implementing prospective public health surveillance systems

Two major elements should be thoroughly considered when implementing a prospective web-based surveillance system. First, one of the potential problems in countries with a high Internet penetration rate is that many people share their personal experiences, perceptions, and distinct individual health conditions via social media, which may not always be a true reflection of the occurrence of a disease activity or an epidemic [3]. In other words, self-reporting and media-driven actions may be a chief confounder of web surveillance systems [3]. Thus, relying solely on data based on lay people’s web queries and post frequency must take into consideration possible inaccurate interpretations.

The majority of the existing web-based surveillance systems work on the premise that disease incidence correlates with the frequency of information-seeking using specific terms [3], which are query data most often analyzed in English. The primary language used to operate these web-based surveillance systems is also English, which limits the frequency of use and monitoring among many people worldwide, and can cause a compatibility problem if the same platforms are used in non-English speaking countries. Repercussions of the language barrier issue will likely affect the accuracy of detecting an outbreak. Several language-related intricacies, including cultural tone, language shifts, and the use of colloquialisms [3] are factors that cannot be easily recognized by technical aspects of web-based surveillance systems as opposed to traditional, conventional surveillance systems maneuvered by human analysts. This is another reason why data accuracy might be heavily affected and constitutes an area for improvement.

Traditional disease surveillance systems are feebly structured but at the same time require high management costs and excessively complex network operation. The most challenging task will be to implement a standardized web-based surveillance system that can be accessed and utilized universally and efficiently at low cost. In high-income, developed countries where the Internet penetration rate is high, the “real-time” feature of these web-based surveillance systems will overcome the limitations of traditional systems with regard to the speed of response and data dissemination. As well, the immediate effect of these systems in developing countries that lack technologies and an efficient public health system will be powerful and innovative. The introduction and amplification of these web-based systems in public health will remedy the shortcomings of traditional systems. Ultimately, the aim is to safely prevent the spread of an infectious disease at early onset by placing timeliness as the utmost priority, so that health consequences of a disease outbreak will be reduced significantly.

## Limitations

This review has several limitations despite employing a systematic review approach and aiming at providing a well-structured overview of web-based infectious disease surveillance systems. Due to limited article accessibility, the literature search was restricted to published articles from a limited number of selected sources. However, as a consequence, we cannot rule out a certain selection and reporting bias in our review. Nevertheless, the here reported work may serve as a good overview and starting point for readers interested in web-based infectious disease surveillance systems. Our hope is that future efforts will further complement and advance our work and provide a continuously updated, more comprehensive and at the same time more detailed picture of the currently existing web-based infectious disease surveillance systems.

## Conclusions

Despite being in a nascent stage, with much modification needed, web-based surveillance systems demonstrate the capacity to complement national traditional surveillance systems [61]. However, the failure of Google Flu Trends shows that continued effort at the national level is required to develop more elaborate web-based surveillance systems. The aim of the present study was to systematically review a compilation of web-based infectious disease surveillance systems to provide the necessary groundwork for developing prospective surveillance systems. Future studies should be diversified and intensified, and involve an expanded scope of research, integration of a wider range of data sources, and the application of advanced methodologies.

## Additional file

Additional file 1: Prisma checklist. (DOC 65 kb)

## Abbreviations

CDC: Centers for disease control and prevention
CIA: Central intelligence agency
EpiSims: Epidemic simulation system
EpiSPIDER: Semantic processing and integration of distributed electronic resources for epidemics
GET WELL: Generating epidemiological trends from web logs, like
GOARN: Global outbreak alert and response network
GPHIN: Global public health intelligence network
MedISys: Medical information system
ProMED-Mail: Program for monitoring emerging diseases
WHO: World health organization
